# Knowledge, Perception, and Attitude of Veterinarians About Q Fever from South Spain

**DOI:** 10.3390/microorganisms13081759

**Published:** 2025-07-28

**Authors:** Francisco Pérez-Pérez, Rafael Jesús Astorga-Márquez, Ángela Galán-Relaño, Carmen Tarradas-Iglesias, Inmaculada Luque-Moreno, Lidia Gómez-Gascón, Juan Antonio De Luque-Ibáñez, Belén Huerta-Lorenzo

**Affiliations:** 1 Competitive Research Unit on Zoonotic and Emerging Diseases (ENZOEM), Department of Animal Health, Faculty of Veterinary Medicine, University of Cordoba, 14071 Cordoba, Spain; v92pepef@uco.es (F.P.-P.); sa1taigc@uco.es (C.T.-I.); sa1lumoi@uco.es (I.L.-M.); v32gogal@uco.es (L.G.-G.); sa2hulob@uco.es (B.H.-L.); 2 Competitive Research Unit on Zoonotic and Emerging Diseases (ENZOEM), Department of Nursing, Pharmacology and Physiotherapy, Pharmacology Area, Faculty of Veterinary Medicine, University of Cordoba, 14071 Cordoba, Spain; agalanr12@gmail.com; 3 Cátedra One Health, Oficial College of Veterinarians, Malaga University, 29017 Malaga, Spain; presidente@colegioveterinariosmalaga.es

**Keywords:** zoonoses, Q Fever, One Health, survey, biosafety

## Abstract

Q Fever is a zoonosis caused by *Coxiella burnetii* that affects domestic and wild ruminants, leading to reproductive disorders. In humans, the disease can manifest with acute and chronic clinical manifestations. Veterinarians, as healthcare professionals in close contact with animals, serve both as the first line of defence in preventing infection at the animal–human interface and as an important sentinel group for the rapid detection of outbreaks. The aim of this study was to assess the knowledge, perception, and attitude of veterinarians in Southern Spain regarding Q Fever. To this end, an online survey was designed, validated, and conducted among veterinarians in the province of Malaga, with a final participation of 97 individuals, predominantly from the private sector (clinic, livestock, agri-food, etc.). The data obtained reflected a general lack of knowledge about the disease, particularly concerning its epidemiology and infection prevention. Regarding perception and attitude, a significant percentage of respondents stated they did not use protective equipment when handling susceptible animals and only sought information about the disease in response to outbreak declarations. The study emphasised the significance of promoting training in zoonotic diseases during and after graduation, the relevance of official channels in occupational risk prevention, and the utility of epidemiological surveys as a tool to identify and address potential gaps in knowledge related to this disease.

## 1. Introduction

Zoonotic agents show complex pathogen–host dynamics and are responsible for over 60% of emerging infectious diseases in humans [1]. This has prompted health authorities to adopt a One Health approach, promoting collaboration across animal, public, and environmental health sectors [2]. Professions involving contact with animals play a crucial role at the human–animal interface, influencing both individual and collective health outcomes [3].

Q Fever, also known as Coxiellosis, is a zoonotic infectious disease caused by the bacterium *Coxiella burnetii* [4], with a global distribution, excluding New Zealand and Antarctica [5]. The number of notifications per 100,000 inhabitants in the European Union was 0.2 for 2019. The highest notification rate (0.7 cases per 100,000 population) was observed in Spain, followed by Romania (0.6), Bulgaria (0.5) and Hungary (0.5) [6]. In the European Union, Spain is the country with the fourth highest number of registered veterinarians (30,000; 0.58 per 1000 inhabitants), surpassed only by Germany, Ukraine, and Italy [7]. Between 2018 and 2023, 52 cases of Q Fever in humans due to zoonotic transmission were detected in the province of Malaga (Illustrious Official College of Veterinarians of Malaga), representing a zoonosis with moderate-to-high prevalence in this location.

Epidemiologically, Q Fever presents two main cycles: a wild cycle, involving ruminants (primarily cervids) and migratory birds, and a domestic cycle, where sheep are the most susceptible species, followed by goats and cattle, although dogs and cats can also contract the infection and act as a source of transmission to humans [8,9]. Both cycles are interconnected through ticks, which serve as reservoir vectors [10]. In southern Spain, the seroprevalence of Q Fever is estimated at 13.88% in sheep and 13.4% in Iberian wild goats [11,12]. The main route of infection and transmission is indirect, through the inhalation of contaminated aerosols or particles. Infected females release large quantities of *Coxiella burnetii* during birth or abortion. Additionally, excretion can occur through faeces, vaginal mucus, urine, and milk. The latter form of excretion could lead to foodborne transmission; however, heat treatments applied to milk and the lack of conclusive evidence mean that the disease is not considered a foodborne risk [13]. Regarding clinical manifestations in animals, Q Fever predominantly impacts the reproductive system, leading to late-term abortions, premature births, retained placentas, and birth of weak offspring. The most characteristic lesions are observed in the placenta, including necrotising placentitis, hemorrhages [14], and bacterial accumulation in the trophoblasts [15]. In humans, Q Fever may present in acute or chronic forms. Acute cases typically involve fever, chills, myalgia, pneumonia, and hepatitis. In its chronic form, the disease may progress to endocarditis and other severe complications, particularly in patients with valvular heart disease, pregnant women, or those who are immunocompromised. It is noteworthy that in the case of the disease in humans, human-to-human transmission is not straightforward and requires external factors such as the presence of mosquito vectors [16]. Diagnosis is based primarily by serological methods (e.g., ELISA, as well as indirect immunofluorescence), while PCR-based techniques are less commonly used. Some reference laboratories are also able to culture *Coxiella burnetii* in cell systems [17]. The Coxevac^®^ vaccine is used in ruminants to reduce abortions and bacterial excretion, with tetracyclines used as treatment [18].

The prevention of Q Fever in humans needs a comprehensive approach encompassing individual, environmental, and public health measures. Immunisation is recommended for high-risk groups such as veterinarians, livestock handlers, and laboratory staff, along with strict hygienic practices including frequent hand washing after contact with animals or rural environments and the use of personal protective equipment (PPE) such as gloves, masks, and specialised clothing. In environmental and occupational settings, it is essential to avoid high-risk areas such as farms and slaughterhouses with infected livestock, mitigate the spread of dust and aerosols, and disinfect contaminated surfaces and clothing. In addition, education of healthcare professionals on the epidemiology of the disease and promotion of clinical suspicion in cases of fever of unknown origin are essential for early detection and accurate differential diagnosis. Together, these strategies substantially reduce the risk of transmission and improve preparedness and response in case of outbreaks of this disease and other conditions with similar epidemiological profiles, such as brucellosis [16].

This disease has a significant impact on public health due to its ability to infect humans, mainly through contact with animals, classifying it as an occupational zoonosis. Although rarely fatal in humans, acute infections can cause fever, fatigue, pneumonia, and hepatitis, while chronic forms may result in endocarditis and other serious complications, especially in immunocompromised individuals or those with valvular heart diseases [16]. Furthermore, Q Fever is included on the list of notifiable diseases by the World Health Organization (WHO) and the World Organization for Animal Health (WOAH).

In the case of Q fever, studies among veterinary students and professionals show a clear relationship between the impact of Q Fever in humans and its presence in animals, highlighting an underestimation of its prevalence due to the fact that most infections are not reported, as they present asymptomatically or with mild flu-like symptoms [19]. In Canada, surveys showed a seroprevalence of 76.2% (95% CI: 52.8–91.8%) in practising veterinarians, 50% (95% CI: 6.8–93.2%) in inactive veterinarians, and 14.3% (95% CI: 0.3–57.9%) in veterinary students [20]. These studies emphasise the need to incorporate zoonotic disease education from the early years of veterinary training and to promote continuous professional development, with special attention to biosafety measures, personal protective equipment, risks associated with animal handling, and specific risks related to preexisting medical conditions, such as immunosuppression or pregnancy [3,21]. Furthermore, health authorities and veterinary faculties are advised to implement surveillance and monitoring programs for the most relevant and emerging zoonoses among students and active veterinarians to enable early case detection and transmission prevention.

The aim of this study was to conduct a cross-sectional survey among veterinarians in the province of Malaga to assess their knowledge, perceptions, and attitudes toward Q Fever.

## 2. Material and Methods

Due to the nature of the information collected, prior approval from the Bioethics Committee of the Junta de Andalucia was required (SICEIA-2024-002752). To comply with the Organic Law on Data Protection and Guarantee of Digital Rights (LODPGDR) 2018 and the General Data Protection Regulation (GDPR) 2022, data anonymisation was maintained throughout the entire research process regarding the study design.

### 2.1. Study Population and Sampling

Between October and December 2024, a cross-sectional survey was conducted among licensed veterinarians in the province of Malaga (covering 27.6% of the total registered veterinarians in Andalusia), remaining open to all professional fields: clinical practice, public health, agriculture, public administrations, laboratories, food companies, and education. This encompassed a study population of 1187 veterinarians. As well as the analysis and presentation of results, the present study was carried out following the STROBE checklist (Strengthening the Reporting of Observational Studies in Epidemiology) [22].

In addition, it is important to note the difference between a licensed veterinarian and a registered veterinarian. A licensed veterinarian refers to a person who has successfully completed an accredited programme of higher education in veterinary medicine at a university. In contrast, a registered veterinarian is one who, after obtaining a veterinary degree, proceeds to register with a Colegio Oficial de Veterinarios—an institutional registration that is a mandatory prerequisite for legal professional practice in Spain [23].

To calculate the sample size, it was estimated that for a confidence level of 95%, a maximum acceptable error of 10%, a standard deviation in the responses of 0.5 (50%), and a study population of 1187 veterinarians (Official College of Veterinarians of Malaga as of 30 September 2024), the sample should consist of 89 participants. This implies that for an expected response rate of 20%, it was necessary to send the survey to at least 445 individuals.

### 2.2. Study Questionnaire

The questionnaire was designed using the online program EUSurvey, developed by the European Commission’s Department for Digital Services. This program enables the creation of a link to facilitate anonymous distribution and participation among the study population. The link was sent to the Veterinary College of Malaga for dissemination via newsletters, the website, and social media, indicating the possibility of forwarding the link to non-member colleagues to expand the sampling framework (“snowball sampling”).

The questionnaire’s development referenced information about Q Fever published on the websites of the WHO, ECDC, WOAH, and Spain’s Ministry of Health. It included single-option and multiple-choice questions. To minimise random correct answers or partial knowledge, questions with more than one correct answer were presented as independent options, allowing participants to select more than one.

Prior to dissemination, the questionnaire underwent validation in two phases. Firstly, it was sent to researchers and professors from the Faculty of Veterinary Medicine in Cordoba, who provided suggestions regarding adequacy, conciseness, and relevance. After revisions, a pilot study was conducted with 20 conveniently available veterinarians in Cordoba. Participants were asked to provide feedback on any difficulties in understanding the survey, and items were revised if at least 15% of respondents reported difficulty with them. Two questions were modified based on this feedback. The final version of the questionnaire consisted of five sections:

- Demographic data (10 items): personal details of the respondent (gender, age, highest level of education, current employment status, professional field, and work location).
- General knowledge of the disease (14 items): etiology, epidemiology, clinical features, diagnosis, and prophylaxis.
- Specific knowledge about the disease in Spain (3 items): aimed at assessing respondents’ understanding of presentation forms, seasonal presentation, and the mandatory reporting of cases.

In both cases, items were scored with ‘1’ point for each correct answer and ‘0’ points if all selected answers were incorrect or the respondent marked “don’t know/no response.”

- Perception of Q Fever (4 items): multiple-choice, single-choice, and numerical rating scale (NRS from 1 to 5 points, with 1 representing the minimum value and 5 the maximum value regarding the availability of reliable information about Q Fever provided by official sources, such as the Ministries of Health and Agriculture.) questions, designed to evaluate participants’ opinions on their training, the quality of information sources, and the occupational zoonotic risk posed by this disease.
- Attitude toward Q Fever (7 items): single-choice and multiple-choice questions, aimed at evaluating attitudes toward the use of personal protective equipment (PPE) and preventive measures, vaccination, early diagnosis, and continued training about the disease.

The estimated average time to complete the questionnaire was 15 min.

### 2.3. Statistical Analysis

A statistical analysis was performed with the IBM SPSS Statistics version 28 program. Based on demographic data, the study population was characterised by calculating the percentages and frequency distributions of the responses obtained for each variable. To assess general and specific knowledge about Q Fever, the following were determined for each item: (1) the percentage of respondents who answered correctly (scores from 1 to 7, depending on the item), (2) the percentage who answered partially, and (3) those who did not answer or answered incorrectly (score of 0). The percentages obtained were statistically compared based on the demographic variables using the non-parametric Kruskal-Walli’s test (α = 0.05). Subsequently, the total achievable score in each section was divided into quartiles, and it was considered that an individual had poor knowledge if their score was below the 50th percentile, good knowledge if it was between the 50th–75th percentiles, and very good knowledge if it exceeded the 75th percentile. The scores of individuals in the different categories were contrasted using the Kruskal-Walli’s rank test (α = 0.05) to assess significant differences based on the demographic characteristics of the respondents.

Finally, the frequency distributions and percentages obtained in the perception and attitude questions were determined and compared based on the demographic characteristics of the respondents, using non-parametric tests (Chi-square, Kruskal-Wallis, and Mann-Whitney U, depending on the nature of the variable) (α = 0.05), applying the Bonferroni correction in the case of pairwise multiple comparisons. In cases where any of the expected frequencies were less than or equal to 4, Fisher’s exact test was used for comparison. The data obtained were graphically represented using the EUSurvey program. As Supplementary Material, the tables generated from the collected data are included, along with the completed survey [Tables S1–S18, Survey S1].

## 3. Results

**Figure 1:**
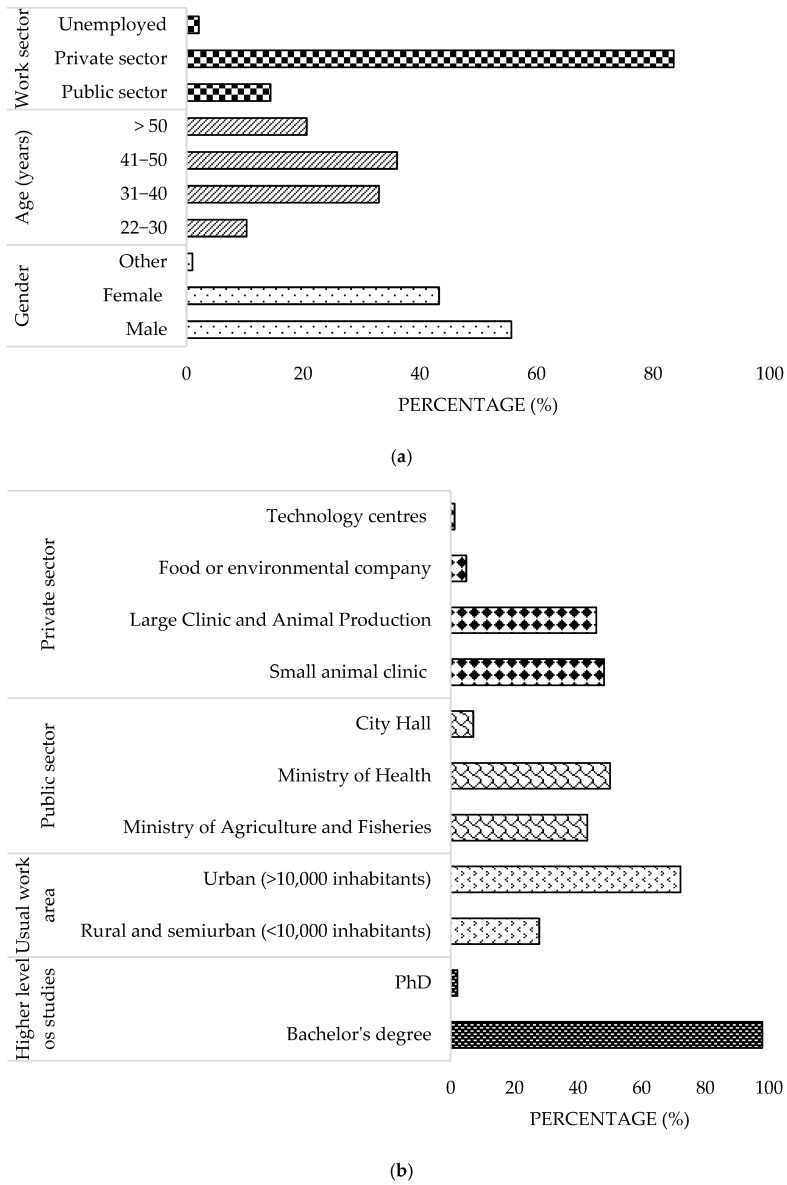
Demographic characteristics of survey veterinary participants from the Province of Malaga (October–December 2024). (**a**) Characterization according to gender, age and work sector. (**b**) Characterization according to level of education and area of work. The frequency distributions and percentages obtained were determined and compared based on the demographic characteristics of the respondents, using non-parametric tests (Chi-square, Fisher’s exact test, Kruskal-Wallis, and Mann-Whitney U, depending on the nature of the variable) (*α* = 0.05), applying the Bonferroni correction in the case of pairwise multiple comparisons.

A total of 97 veterinarians participated, who answered all the questions in the survey (necessary requirement to complete the questionnaire), of whom 69% were between 31 and 50 years old, 55.7% were male, and 97.9% held a degree in Veterinary Medicine. Of the participants, 72.2% worked in urban areas and 83.5% were employed in the private sector, primarily in small and large animal clinics. In the public sector (14.4%), 92.9% worked in departments related to Agriculture, Fisheries, and Health (Figure 1a,b).

**Figure 2:**
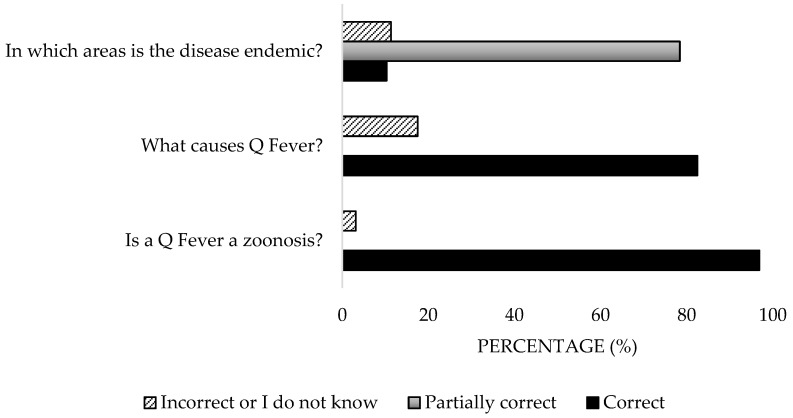
Knowledge about ethology and spread of the disease of survey veterinary participants from the Province of Malaga (October–December 2024). The frequency distributions and percentages obtained were determined and compared based on the demographic characteristics of the respondents, using non-parametric tests (Chi-square, Fisher’s exact test, Kruskal-Wallis, and Mann-Whitney U, depending on the nature of the variable) (*α* = 0.05), applying the Bonferroni correction in the case of pairwise multiple comparisons.

Most of the participants (96.9%) were aware of the zoonotic nature of the disease, and 82.47% recognised its bacterial origin (Figure 2). However, 10.3% mistakenly identified the vector as the etiological agent. Regarding endemic distribution, only 10.3% answered all options correctly, while 78.4% provided partially correct responses. As for reservoirs, 92.8% identified domestic ruminants, but only 9.3% recognised birds and deer as wild reservoirs. Significant differences were detected in the work sector (*p* = 0.038).

**Figure 3:**
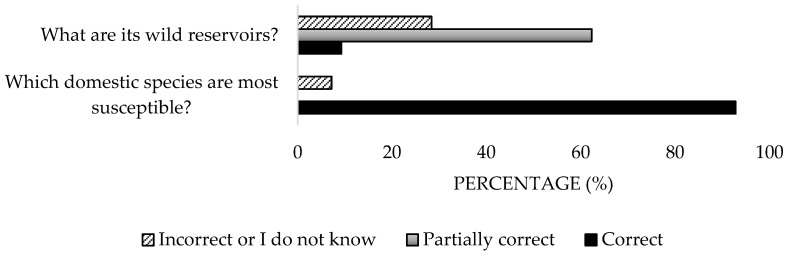
Knowledge about reservoirs and modes of transmission of the disease of survey veterinary participants from the Province of Malaga (October–December 2024). The frequency distributions and percentages obtained were determined and compared based on the demographic characteristics of the respondents, using non-parametric tests (Chi-square, Fisher’s exact test, Kruskal-Wallis, and Mann-Whitney U, depending on the nature of the variable) (*α* = 0.05), applying the Bonferroni correction in the case of pairwise multiple comparisons.

**Figure 4:**
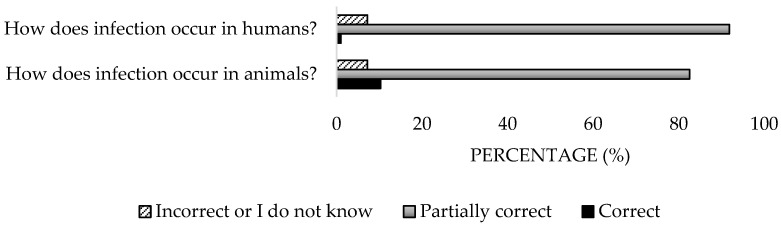
Knowledge about modes of transmission of the disease of survey veterinary participants from the Province of Malaga (October–December 2024). The frequency distributions and percentages obtained were determined and compared based on the demographic characteristics of the respondents, using non-parametric tests (Chi-square, Fisher’s exact test, Kruskal-Wallis, and Mann-Whitney U, depending on the nature of the variable) (*α* = 0.05), applying the Bonferroni correction in the case of pairwise multiple comparisons.

**Figure 5:**
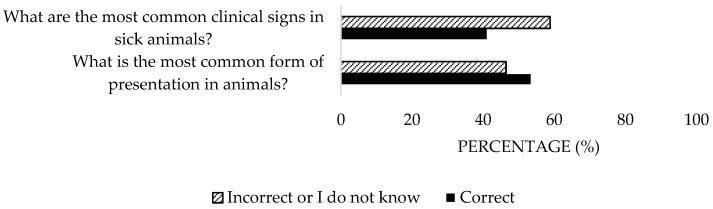
Knowledge about forms of presentation and clinical signs in animals of the disease of survey veterinary participants from the Province of Malaga (October–December 2024). The frequency distributions and percentages obtained were determined and compared based on the demographic characteristics of the respondents, using non-parametric tests (Chi-square, Fisher’s exact test, Kruskal-Wallis, and Mann-Whitney U, depending on the nature of the variable) (*α* = 0.05), applying the Bonferroni correction in the case of pairwise multiple comparisons.

**Figure 6:**
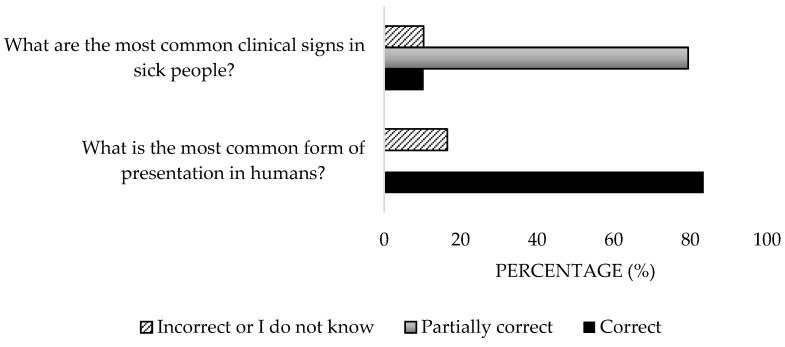
Knowledge about forms of presentation and clinical signs in humans of the disease of survey veterinary participants from the Province of Malaga (October–December 2024). The frequency distributions and percentages obtained were determined and compared based on the demographic characteristics of the respondents, using non-parametric tests (Chi-square, Fisher’s exact test, Kruskal-Wallis, and Mann-Whitney U, depending on the nature of the variable) (*α* = 0.05), applying the Bonferroni correction in the case of pairwise multiple comparisons.

A high percentage of the participants (84.5%) identified tick bites and the inhalation of contaminated aerosols as transmission routes (Figure 3), but only 15.3% associated human transmission with contact with animals (Figure 4). Regarding clinical manifestations in animals, 53.6% were aware of their asymptomatic nature, but 58.8% incorrectly responded about clinical signs (Figure 5). Only 41.2% accurately identified reproductive disorders. For humans, the majority (83.5%) understood that the most common presentation is asymptomatic, yet only 10.3% identified all clinical signs (Figure 6). Significant differences were detected in the private sector (*p* = 0.04).

**Figure 7:**
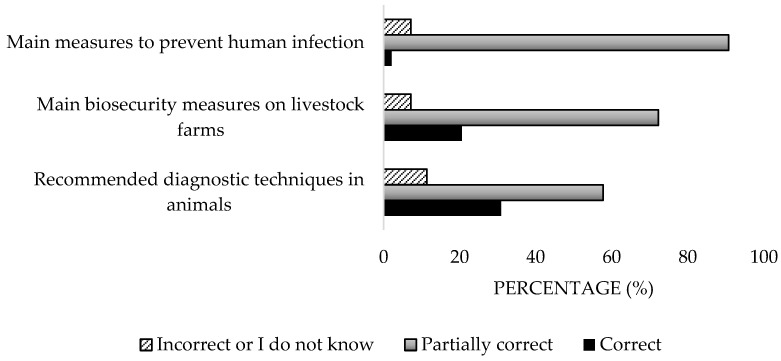
Knowledge about diagnosis and prevention of the disease of survey veterinary participants from the Province of Malaga (October–December 2024). The frequency distributions and percentages obtained were determined and compared based on the demographic characteristics of the respondents, using non-parametric tests (Chi-square, Fisher’s exact test, Kruskal-Wallis, and Mann-Whitney U, depending on the nature of the variable) (*α* = 0.05), applying the Bonferroni correction in the case of pairwise multiple comparisons.

In terms of diagnosis, the majority chose PCR, disregarding ELISA, which is recommended by the WOAH (2018) [17]. Only 20.6% identified all the necessary biosafety measures for livestock farms, with the most selected being faeces treatment (process to eliminate pathogenic microorganisms present in animal excrement in order to prevent disease transmission and protect the environment), desinfection, vaccination, and tick control (Figure 7). Regarding human prevention, 90.7% provided partially correct responses, with the most selected measures being the use of protective clothing and tick control. Additionally, only 14.4% mentioned vaccination, and 17.5% identified quarantine as a preventive measure. Significant differences were detected in the private sector (*p* = 0.002).

**Figure 8:**
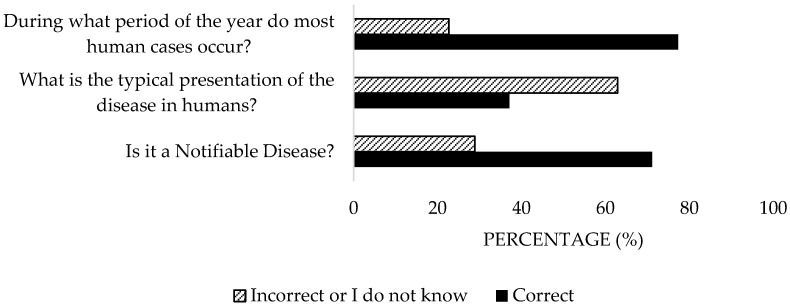
Knowledge of the disease in Spain of survey veterinary participants from the Province of Malaga (October–December 2024). The frequency distributions and percentages obtained were determined and compared based on the demographic characteristics of the respondents, using non-parametric tests (Chi-square, Fisher’s exact test, Kruskal-Wallis, and Mann-Whitney U, depending on the nature of the variable) (*α* = 0.05), applying the Bonferroni correction in the case of pairwise multiple comparisons.

A remarkable percentage of the participants (71.1%) was aware of the classification of Q Fever as a Notifiable Disease (ND) (Figure 8). However, the majority (62.9%) answered incorrectly regarding its presentation in humans. Nevertheless, 77.3% correctly associated the lambing season with peaks in cases. Significant differences were detected in the private sector (*p* = 0.04).

**Figure 9:**
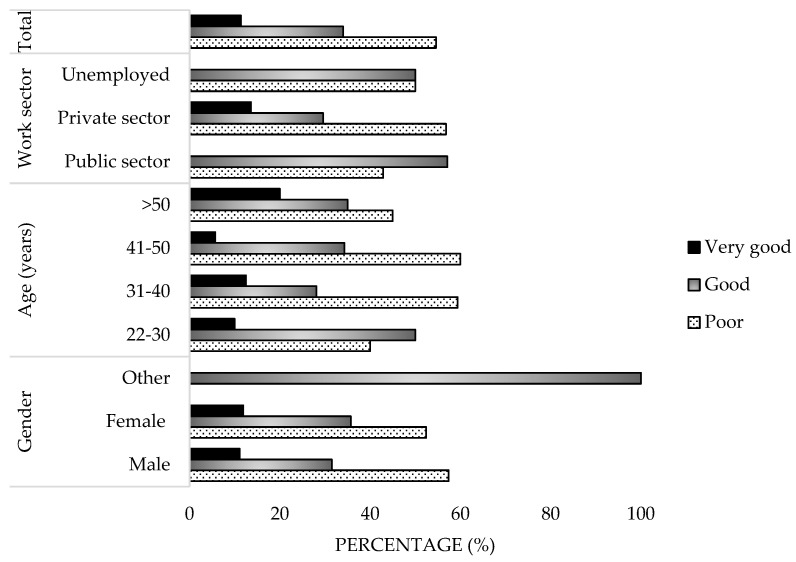
Classification of knowledge of survey veterinary participants from the Province of Malaga (October–December 2024). The frequency distributions and percentages obtained were determined and compared based on the demographic characteristics of the respondents, using non-parametric tests (Chi-square, Fisher’s exact test, Kruskal-Wallis, and Mann-Whitney U, depending on the nature of the variable) (*α* = 0.05), applying the Bonferroni correction in the case of pairwise multiple comparisons.

**Figure 10:**
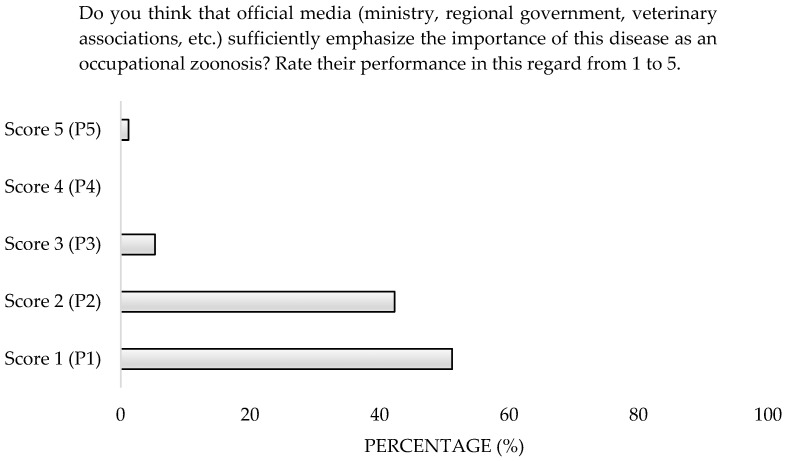
Perception of the information received through official channels about Q Fever of survey veterinary participants from the Province of Malaga (October–December 2024). The frequency distributions and percentages obtained were determined and compared based on the demographic characteristics of the respondents, using non-parametric tests (Chi-square, Fisher’s exact test, Kruskal-Wallis, and Mann-Whitney U, depending on the nature of the variable) (*α* = 0.05), applying the Bonferroni correction in the case of pairwise multiple comparisons.

**Figure 11:**
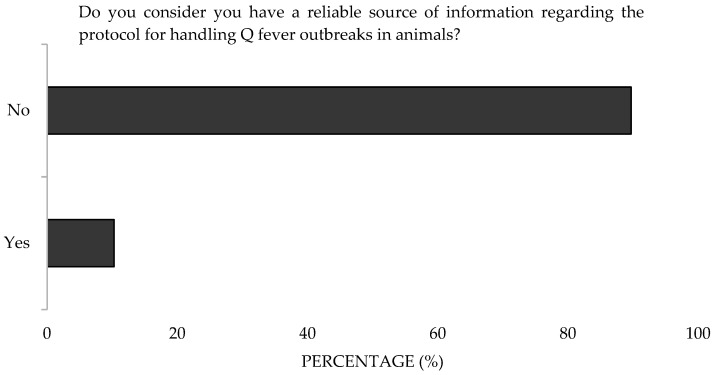
Perception of the reliability of information sources about Q Fever of survey veterinary participants from the Province of Malaga (October–December 2024). The frequency distributions and percentages obtained were determined and compared based on the demographic characteristics of the respondents, using non-parametric tests (Chi-square, Fisher’s exact test, Kruskal-Wallis, and Mann-Whitney U, depending on the nature of the variable) (*α* = 0.05), applying the Bonferroni correction in the case of pairwise multiple comparisons.

More than half of the participants (54.6%) demonstrated Poor Knowledge, while 34% demonstrated Good Knowledge and 11.4% Very Good Knowledge (Figure 9). There were no differences observed based on gender, age, or workplace environment; however, participants aged 22–30 scored higher, with 50% demonstrating Good Knowledge. Additionally, 93.5% considered official information regarding Q Fever as an occupational zoonosis to be insufficient (Figure 10). Moreover, 89.7% lacked a reliable source for protocols to address outbreaks (Figure 11).

**Figure 12:**
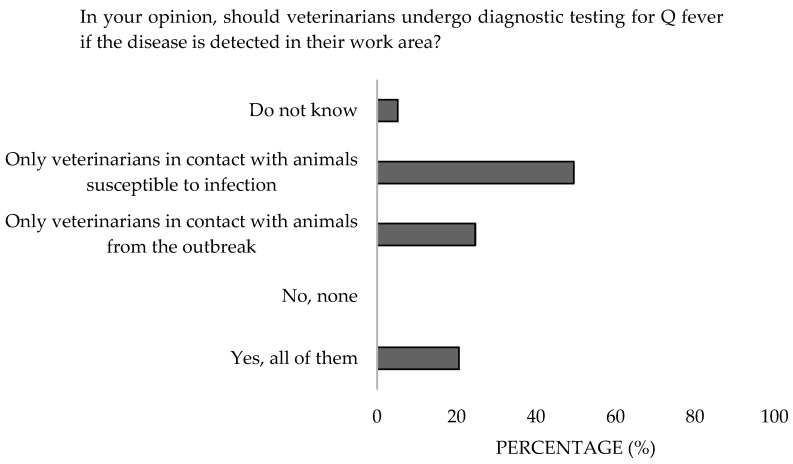
Perception of the performance of diagnostic tests of survey veterinary participants from the Province of Malaga (October–December 2024). The frequency distributions and percentages obtained were determined and compared based on the demographic characteristics of the respondents, using non-parametric tests (Chi-square, Fisher’s exact test, Kruskal-Wallis, and Mann-Whitney U, depending on the nature of the variable) (*α* = 0.05), applying the Bonferroni correction in the case of pairwise multiple comparisons.

**Figure 13:**
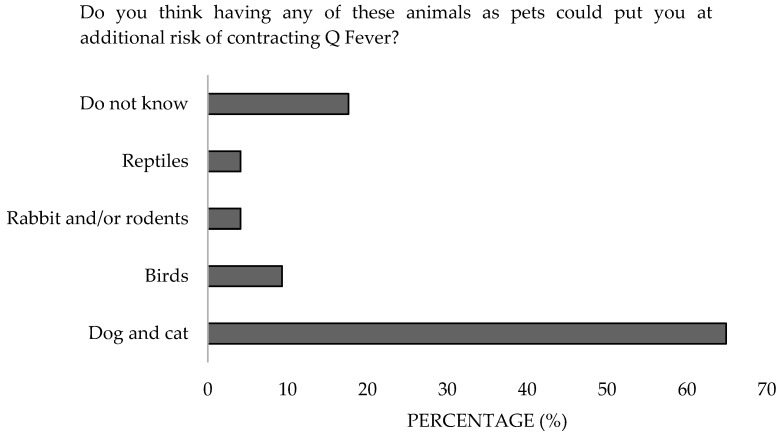
Perception of the risk of having pets of survey veterinary participants from the Province of Malaga based on several demographic variables (October–December 2024). The frequency distributions and percentages obtained were determined and compared based on the demographic characteristics of the respondents, using non-parametric tests (Chi-square, Fisher’s exact test, Kruskal-Wallis, and Mann-Whitney U, depending on the nature of the variable) (*α* = 0.05), applying the Bonferroni correction in the case of pairwise multiple comparisons.

Regarding the diagnosis of exposed veterinarians, 49.5% believed that only those working with susceptible animals should undergo testing, and 64.9% associated dogs and cats with transmission, but only 9.3% included birds (Figure 12). Significant differences were detected in the work sector (*p* = 0.04). Additionally, 17.6% were unaware of the involvement of pets (Figure 13). Significant differences were detected in the work sector (*p* = 0.01) and the knowlegde (*p* = 0.02).

**Figure 14:**
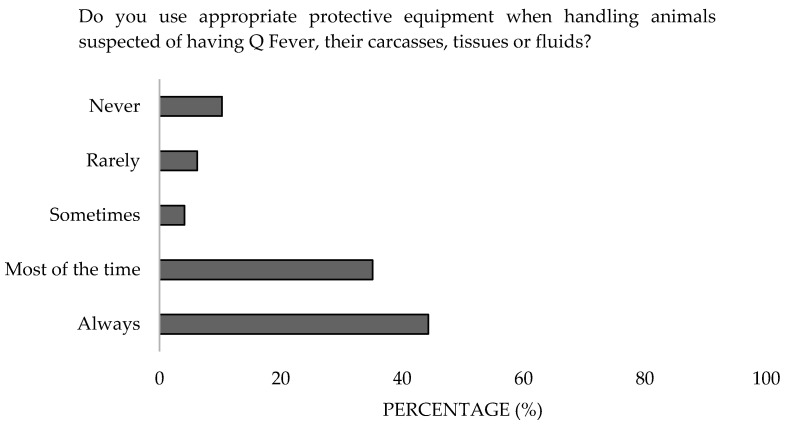
Attitude toward the use of protective equipment of survey veterinary participants from the Province of Malaga (October–December 2024). The frequency distributions and percentages obtained were determined and compared based on the demographic characteristics of the respondents, using non-parametric tests (Chi-square, Fisher’s exact test, Kruskal-Wallis, and Mann-Whitney U, depending on the nature of the variable) (*α* = 0.05), applying the Bonferroni correction in the case of pairwise multiple comparisons.

**Figure 15:**
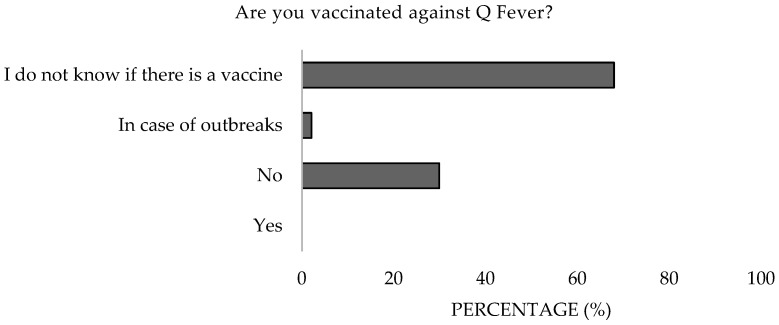
Attitude toward vaccination status against Q Fever of survey veterinary participants from the Province of Malaga (October–December 2024). The frequency distributions and percentages obtained were determined and compared based on the demographic characteristics of the respondents, using non-parametric tests (Chi-square, Fisher’s exact test, Kruskal-Wallis, and Mann-Whitney U, depending on the nature of the variable) (*α* = 0.05), applying the Bonferroni correction in the case of pairwise multiple comparisons.

**Figure 16:**
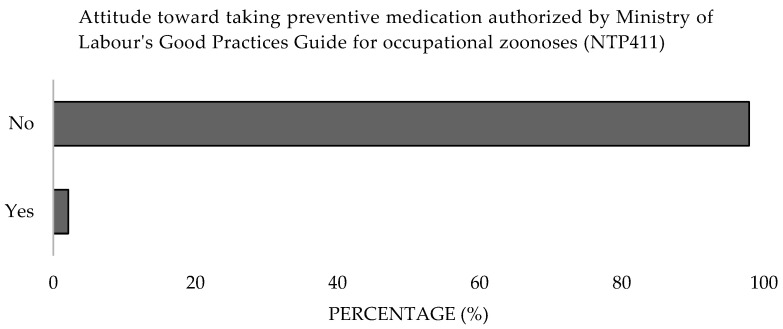
Attitude toward taking preventive medication authorised by Ministry of Labour’s Good Practices Guide for occupational zoonoses (NTP411) of survey veterinary participants from the Province of Malaga (October–December 2024). The frequency distributions and percentages obtained were determined and compared based on the demographic characteristics of the respondents, using non-parametric tests (Chi-square, Fisher’s exact test, Kruskal-Wallis, and Mann-Whitney U, depending on the nature of the variable) (*α* = 0.05), applying the Bonferroni correction in the case of pairwise multiple comparisons.

Concerning the use of protective equipment, 79.4% reported always or almost always using it, while 16.5% used it rarely (Figure 14), with the latter group notably comprising government employees and small animal clinicians (*α* = 0.05). In terms of vaccination, 97.9% were neither vaccinated nor aware of the vaccine, except for 2.1% who stated they would get vaccinated in the event of an outbreak (Figure 15). Additionally, 97.9% were unaware of the possibility of chemoprophylaxis for exposed professionals, as outlined in the Good Practices Guide of the Ministry of Labor and Social Affairs of Spain (NTP 411) [24]. Moreover, only 1% took preventive medication when there was a possibility of contracting a zoonotic disease (Figure 16). Significant differences were detected in the work sector (*p* = 0.001).

**Figure 17:**
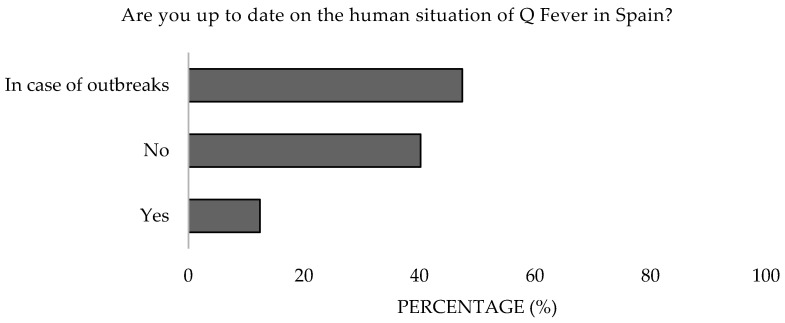
Attitude toward staying informed about Q Fever in humans of survey veterinary participants from the Province of Malaga (October–December 2024). The frequency distributions and percentages obtained were determined and compared based on the demographic characteristics of the respondents, using non-parametric tests (Chi-square, Kruskal-Wallis, and Mann-Whitney U, depending on the nature of the variable) (*α* = 0.05) applying the Bonferroni correction in the case of pairwise multiple comparisons.

**Figure 18:**
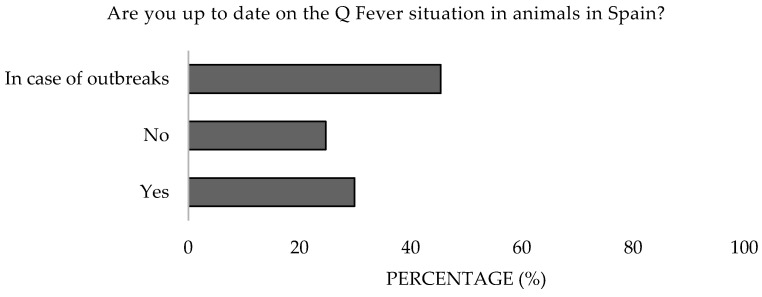
Attitude toward staying informed about Q Fever in animals of survey veterinary participants from the Province of Malaga (October–December 2024). The frequency distributions and percentages obtained were determined and compared based on the demographic characteristics of the respondents, using non-parametric tests (Chi-square, Kruskal-Wallis, and Mann-Whitney U, depending on the nature of the variable) (*α* = 0.05), applying the Bonferroni correction in the case of pairwise multiple comparisons.

Moreover, 24.7% and 40.2% of participants admitted to not being up to date with Q Fever in animals and humans in the Spanish context. The 31–40 age group demonstrated greater interest (Figure 17 and Figure 18). Significant differences were detected in the age (*p* = 0.047 and *p* = 0.005).

## 4. Discussion

The survey reveals a worrying deficiency in epidemiological knowledge and preventive practices in relation to Q fever among veterinary professionals in Malaga. These results were expected and are attributable to the limited diagnostic capacity of the disease. Most respondents did not identify direct contact with infected animals as an important route of zoonotic transmission, and more than 60% were unaware of the clinical manifestations of the disease in humans, despite the fact that Spain is the leading EU country in terms of reported cases. In addition, almost 90% lacked reliable outbreak response protocols, and regular updates on the epizootic and endemic status of Q fever in animals and humans were remarkably insufficient, an expected result due to the lack of consensus on operational protocols at European level. More than half of the participants did not systematically use personal protective equipment (PPE) when handling animals potentially infected with *Coxiella burnetii*, which increased the risk of transmission, a result that surprised the authors due to the fundamental role of PPE as a barrier against transmission.

Finally, the overall level of awareness among veterinarians was classified as Poor, a result expected due to the lack of awareness of the disease among respondents, which underlines the urgent need for improved continuing education initiatives by professional associations and proactive engagement of the veterinary community.

It is a well-established fact that veterinarians are at a higher risk of infection by zoonotic agents compared to the general population, with a significantly higher seroprevalence for pathogens such as *Brucella* spp., *Coxiella burnetii*, *Chlamydia psittaci*, *Bartonella henselae*, methicillin-resistant *Staphylococcus aureus* (MRSA), *Streptococcus suis* type 2, Hepatitis E, or *Toxocara canis* [21]. Furthermore, among the emerging zoonotic agents identified in recent decades (2007–2015), several pathogens with high occupational transmission have been reported, including the viruses of West Nile Fever, Rift Valley Fever, and Crimean-Congo Hemorrhagic Fever, as well as *Coxiella burnetii*, *S. suis*, and the clonal complex 398 of methicillin-resistant *S. aureus* [25]. The frequency of veterinarians who acknowledge having contracted a zoonotic disease ranges from 16.7% to 64%, depending on the geographical area [26,27]. In this context, veterinarians can serve as an important source of infection for other individuals and animals and act as sentinels for the early detection of emerging zoonoses.

Nevertheless, very few studies have been conducted in Europe to assess the actual knowledge, perception, and attitude of veterinarians regarding Q Fever [28], with our research being the first to address this issue in Spain. The average age of veterinarians in Spain is 42 years, with a gender distribution of 36% men and 63% women. In Andalusia, however, the latest data published by the National Institute of Statistics (INE) indicate a gender distribution similar to that obtained in our research sample (50.8% men and 49.2% women) [29].

According to the results obtained in this study, the majority of participants (54.6%) had a rather poor understanding of Q Fever, particularly regarding its transmission to humans through contact with animals, their carcasses, tissues, or fluids, as well as the ingestion of contaminated water and food, which poses a significant risk of infection. These findings align with those reported by Bañuls et al. (2024) [30] concerning Crimean-Congo hemorrhagic fever (CCHF) in Spain (65.2% Poor, 24.4% Good, and 10.4% Very Good) and by Crist et al. (2022) [31] in Illinois (USA) for other tick-borne diseases.

Additionally, 17.6% were unaware of the role of pets (dogs and cats) as potential sources of infection [9,32], despite the fact that more than half of the respondents (9/17 = 52.9%) were clinicians specialising in small and large animals. This aligns with the findings of Sellens et al. (2016) [33], whose study concluded that many Australian veterinary clinicians were unaware of the risk posed by pets as a source of Q Fever infection due to the belief that only ruminant livestock can transmit the disease to humans.

With respect to their ability to identify the clinical manifestations of this disease in animals, only 41.2% correctly recognised the reproductive disorders typical of Q Fever, a significantly lower percentage than that reported by Winter and Campe (2022) [34] in Germany (72.6%). No significant differences were observed between regions or work environments (clinical practice, livestock farming, food industry, etc.), although it would have been expected that veterinarians specialising in large animals would demonstrate greater knowledge. Regarding human health, the majority of participants (62.9%) admitted to not being familiar with the clinical presentation of the disease, which contrasts with its endemic nature in Spain [1] and the fact that Spain is the European Union country—excluding the United Kingdom—with the highest number of annually reported cases: 303 out of 719 cases in 2023, with an incidence rate of 0.64 per 100,000 inhabitants (European average: 0.17 cases per 100,000 inhabitants), second only to Hungary (incidence rate of 0.69) [6]. Data published by the Spanish Epidemiological Surveillance Network for 2023 confirm an increasing trend compared to 2021 and 2022, with a total of 519 autochthonous cases and three imported cases reported. Additionally, 81.4% of respondents did not recognised symptoms such as fatigue, pneumonia, and endocarditis, which may be attributed to limited training in public health during and after their studies and/or the clinical variability of the acute form of the disease depending on the geographical region [35].

The surveillance of Q Fever in the European Union is mandatory in 24 countries (including Spain), voluntary in France, and nonexistent in Austria. However, nearly 30% of veterinarians were unaware of the reporting requirement [36], a percentage very similar to that reported by Bañuls et al. (2024) [30] for CCHF (70.6%). Regarding their perception of occupational risk and their attitude toward prevention, almost 90% of respondents highlighted the limited information provided by official communication channels (ministries, professional associations, etc.) and the lack of a reliable source for consultation on the response protocol in the event of an outbreak in animals. As for the protective measures used, although the majority reported routinely using personal protective equipment (PPE), they did not usually complement it with periodic serological screenings, medical prophylaxis, or vaccination. Similar results were reported at the national level by Bañuls et al. (2024) [30] for zoonoses in general. Finally, it is noteworthy that only 17.5% of veterinarians identified animal quarantine or systematic pesticide treatment as preventive measures for human infection, both of which are fundamental given the zoonotic nature of this disease [1,37].

In general, proper hygiene and the use of personal protective equipment (PPE) are considered important measures to prevent infection by *Coxiella burnetii* [19]. However, these measures may not be sufficient. Following the culling campaign conducted in the Netherlands between 2009 and 2010 to control Q Fever outbreaks in goat herds, 17.5% of workers seroconverted despite their experience in using PPE, which led to the implementation of vaccination for this at-risk group [38]. Although the Australian vaccine is contraindicated for seropositive individuals, it could be a viable option for seronegative veterinary students and professionals, particularly those with a medical history that increases their risk. In any case, when presenting with symptoms such as fever, fatigue, headache, or general discomfort, veterinarians working with livestock should inform their physician of their potencial occupational exposure to facilitate early diagnosis of *Coxiella burnetii* infection and prevent the development of more severe chronic forms, such as valvular endocarditis [3].

The results of our study support the recommendations made by other authors regarding the need to review and improve the content on zoonotic diseases in veterinary degree programs, promote continuous education among healthcare professionals, and develop risk management plans through official channels [39]. In this regard, it is important to highlight the Standard Operating Procedures for Biosafety manual, developed by the Biosafety Working Group of the Faculty of Veterinary Medicine at the University of Liège (2019) [40], under the direction of Professor Saegerman. This manual outlines general and specific protocols related to the handling of animal species and various professional fields, tailored to different user profiles (students, students with disabilities, veterinarians, visitors, staff). Similarly, the development and publication of books or manuals on protection, occupational health, and control measures against zoonoses are of particular interest [41,42].

## 5. Conclusions

As the findings of this study demonstrate, veterinarians present an excellent opportunity for the monitoring of endemic zoonoses such as Q Fever. Therefore, it is essential to strengthen training programs and health education initiatives within the sector, particularly in the case of zoonoses, where a lower level of knowledge has been observed among these professionals.

Additionally, the study highlighted the importance of epidemiological surveys as a tool for identifying and addressing potential knowledge gaps related to this disease.

## 6. Limitations

Due to the cross-sectional nature of the survey, the sample size in some demographic categories was small, which could have influenced the detection of differences as significant (*α* = 0.05) despite being irrelevant.

## Data Availability

The original contributions presented in this study are included in the article. Further inquiries can be directed to the corresponding author.

## Supplementary Materials

The following supporting information can be downloaded at: https://www.mdpi.com/article/10.3390/microorganisms13081759/s1, Table S1: Demographic characteristics of survey veterinary participants from the Province of Malaga (October–December 2024); Table S2: Knowledge about ethology and spread of the disease; Table S3: Knowledge about reservoirs and modes of transmission of the disease; Table S4: Knowledge about modes of transmission of the disease; Table S5: Knowledge about forms of presentation and clinical signs in animals of the disease; Table S6: Knowledge about forms of presentation and clinical signs in humans of the disease; Table S7: Knowledge about diagnosis and prevention of the disease; Table S8: Knowledge of the disease in Spain; Table S9: Classification of knowledge; Table S10: Perception of the information received through official channels about Q Fever; Table S11: Perception of the reliability of information sources about Q Fever; Table S12: Perception of the performance of diagnostic tests; Table S13: Perception of the risk of having pets; Table S14: Attitude toward the use of protective equipment; Table S15: Attitude toward vaccination status against Q Fever; Table S16: Attitude toward taking preventive medication authorized by Ministry of Labour’s Good Practices Guide for occupational zoonoses (NTP411); Table S17: Attitude toward staying informed about Q Fever in humans; Table S18: Attitude toward staying informed about Q Fever in animals; Survey S1: Knowledge, perception, and attitude toward Q Fever among veterinarians in the province of Malaga.
