# Supplementary material for: Knowledge, Perception, and Attitude of Veterinarians About Q Fever from South Spain
Source: Microorganisms. 2025 Jul 28;13(8):1759. doi: 10.3390/microorganisms13081759 (PMC12388058; doi:10.3390/microorganisms13081759)
Supplement: Supplementary file 1 [file microorganisms-13-01759-s001.zip › microorganisms-3709445-Supplementary material.pdf]

Supplementary material

Supplementary A. Complementary tables

Table S1. Demographic characteristics of survey veterinary participants from the Province of Malaga (October-December 2024). The frequency distributions and percentages obtained were determined and compared based on the demographic characteristics of the respondents, using non-parametric tests (Chi-square, Fisher's exact test, Kruskal-Wallis, and Mann-Whitney U, depending on the nature of the variable) ($\alpha = 0.05$), applying the Bonferroni correction in the case of pairwise multiple comparisons.

**Total participants (n=97)**	**n (Percentage %)**	**$CI_{95\%}$**
**Gender**		
Male	54 (55.7%)	45.8–65.6%
Female	42 (43.3%)	33.4–53.2%
Other	1 (1%)	0-3%
**Age (years)**		
22 - 30	10 (10.3%)	4.3–16.4%
31 - 40	32 (33%)	23.6–42.4%
41 - 50	35 (36.1%)	26.5–45.6%
Over 50	20 (20.6%)	12.6–28.7%
**Higher level of studies**		
Bachelor’s degree in veterinary medicine	95 (97.9%)	95.1–100%
PhD in Veterinary Medicine	2 (2.1%)	0–4.9%
**Another higher level of studies**		
Yes	8 (8.3%)	2.8–7.13%
No	89 (91.7%)	86.3–97.2%
**Usual work area**		
Rural and semi-urban (< 10,000 inhabitants)	27(27.8%)	18.9–36.8%
Urban (> 10,000 inhabitants)	70 (72.2%)	63.3–81.1%
**Work sector**		
Public sector	14 (14.4%)	7.4–21.4%
Private sector	81 (83.5%)	76.1–90.9%
Unemployed	2 (2.1%)	0–4.9%
**Public sector (n=14)**		
Ministry of Agriculture and Fisheries	6 (42.9%)	16.9–68.8%
Ministry of Health	7 (50%)	23.8–76.2%
City Hall	1 (7.1%)	0–20.6%
**Private sector (n=81)**		
Small animal clinic only	39 (48.2%)	37.3-59%
Large Clinic and Animal Production	37 (45.7%)	34.8–56.5%
Food or environmental company	4 (4.9%)	0.2–9.7%
Technology centres and laboratories	1 (1.2%)	0–3.6%

Table S2. Knowledge about ethology and spread of the disease of survey veterinary participants from the Province of Malaga (October-December 2024). The frequency distributions and percentages obtained were determined and compared based on the demographic characteristics of the respondents, using non-parametric tests (Chi-square, Fisher's exact test, Kruskal-Wallis, and Mann-Whitney U, depending on the nature of the variable) (α = 0.05), applying the Bonferroni correction in the case of pairwise multiple comparisons.

**Total participants (n=97)**				
**Question**	**Correct n (%)**	**Partially correct n (%)**	**Incorrect or I do not know n (%)**	*P* Value
**Is Q Fever a zoonosis?**	**94 (96.9)**	**-**	**3 (3.1)**	
Work area				
Rural-semi-urban (n=27)	26 (96.3)	-	1 (3.7)	1.00
Urban (n=70)	68 (97.1)	-	2 (2.9)	
Work sector				
Public sector (n=14)	14 (100)	-	0 (0)	
Private sector (n=81)	78 (96.3)	-	3 (3.7)	0.73
Unemployed (n=2)	2 (100)	-	0 (0)	
Private sector				
Small animals (n=39)	38 (97.4)	-	1 (2.6)	
Large animals (n=37)	36 (97.3)	-	1 (2.7)	0.14
Company (n=4)	3 (75)	-	1 (25)	
Technology centre (n=1)	1 (100)	-	0 (0)	
**What causes Q Fever?**	**80 (82.5)**	**-**	**17 (17.5)**	
Work area				
Rural-semi-urban (n=27)	23 (85.2)	-	4 (14.8)	0.77
Urban (n=70)	57 (81.4)	-	13 (18.6)	
Work sector				
Public sector (n=14)	11 (78.6)	-	3 (21.4)	
Private sector (n=81)	68 (84)	-	13 (16)	0.42
Unemployed (n=2)	1 (50)	-	1 (50)	
Private sector				
Small animals (n=39)	33 (84.6)	-	6 (15.4)	
Large animals (n=37)	31 (83.8)	-	6 (16.2)	0.93
Company (n=4)	3 (75)	-	1 (25)	
Technology centre (n=1)	1 (100)	-	0 (0)	
**In which areas is the disease endemic?**	**10 (10.3)**	**76 (78.4)**	**11 (11.3)**	
Work area				
Rural-semi-urban (n=27)	4 (14.5)	20 (74.1)	3 (11.4)	0.91
Urban (n=70)	6 (8.6)	56 (80)	8 (11.4)	
Work sector				
Public sector (n=14)	0 (0)	11 (78.6)	3 (21.4)	
Private sector (n=81)	10 (12.4)	63 (77.8)	8 (9.8)	0.51
Unemployed (n=2)	0 (0)	2 (100)	0 (0)	
Private sector				
Small animals (n=39)	4 (10.3)	31 (79.4)	4 (10.3)	
Large animals (n=37)	6 (16.2)	29 (78.4)	2 (5.4)	0.51
Company (n=4)	0 (0)	2 (50)	2 (50)	
Technology centre (n=1)	0 (0)	1 (100)	0 (0)	

Table S3. Knowledge about reservoirs and modes of transmission of the disease of survey veterinary participants from the Province of Malaga (October-December 2024). The frequency distributions and percentages obtained were determined and compared based on the demographic characteristics of the respondents, using non-parametric tests (Chi-square, Fisher's exact test, Kruskal-Wallis, and Mann-Whitney U, depending on the nature of the variable) (α = 0.05), applying the Bonferroni correction in the case of pairwise multiple comparisons.

Total participants (n=97)				
**Question**	**Correct n (%)**	**Partially correct n (%)**	**Incorrect or I do not know n (%)**	*P* Value
**Which domestic species are most susceptible?**	**90 (92.8)**	**-**	**7 (7.2)**	
Work area				
Rural-semi-urban (n=27)	26 (96.3)	-	1 (3.7)	0.66
Urban (n=70)	64 (91.4)	-	6 (8.6)	
Work sector				
Public sector (n=14)	14 (100)	-	0 (0)	
Private sector (n=81)	75 (92.6)	-	6 (7.4)	**0.038**
Unemployed (n=2)	1 (50)	-	1 (50)	
Private sector				
Small animals (n=39)	36 (92.3)	-	3 (7.7)	
Large animals (n=37)	35 (94.6)	-	2 (5.4)	0.55
Company (n=4)	3 (75)	-	1 (25)	
Technology centre (n=1)	1 (100)	-	0 (0)	
**What are its wild reservoirs?**	**9 (9.3)**	**60 (62.3)**	**28 (28.4)**	
Work area				
Rural-semi-urban (n=27)	3 (11.1)	16 (59.3)	8 (29.6)	0.87
Urban (n=70)	6 (8.6)	45 (64.3)	19 (27.1)	
Work sector				
Public sector (n=14)	0 (0)	9 (64.3)	5 (35.7)	
Private sector (n=81)	9 (11.1)	51 (63)	21 (25.9)	0.62
Unemployed (n=2)	0 (0)	1 (50)	1 (50)	
Private sector				
Small animals (n=39)	3 (7.7)	25 (64.1)	11 (28.2)	
Large animals (n=37)	6 (16.2)	22 (59.5)	9 (24.3)	0.85
Company (n=4)	0 (0)	3 (75)	1 (25)	
Technology centre (n=1)	0 (0)	1 (100)	0 (0)	

Table S4. Knowledge about modes of transmission of the disease of survey veterinary participants from the Province of Malaga (October-December 2024). The frequency distributions and percentages obtained were determined and compared based on the demographic characteristics of the respondents, using non-parametric tests (Chi-square, Fisher's exact test, Kruskal-Wallis, and Mann-Whitney U, depending on the nature of the variable) (α = 0.05), applying the Bonferroni correction in the case of pairwise multiple comparisons.

**Total participants (n=97)**				
**Question**	**Correct n (%)**	**Partially correct n (%)**	**Incorrect or I do not know n (%)**	*P* Value
**How does infection occur in animals?**	**10 (10.3)**	**80 (82.5)**	**7 (7.2)**	
Work area				
Rural-semi-urban (n=27)	1 (3.7)	25 (92.6)	1 (3.7)	0.57
Urban (n=70)	9 (12.9)	55 (78.6)	6 (8.5)	
Work sector				
Public sector (n=14)	0 (0)	13 (92.9)	1 (7.1)	
Private sector (n=81)	9 (11.1)	66 (81.5)	6 (7.4)	0.15
Unemployed (n=2)	1 (50)	1 (50)	0 (0)	
Private sector				
Small animals (n=39)	6 (15.6)	30 (77)	3 (7.4)	
Large animals (n=37)	3 (8.1)	32 (86.5)	2 (5.4)	0.81
Company (n=4)	0 (0)	3 (75)	1 (25)	
Technology centre (n=1)	0 (0)	1 (100)	0 (0)	
**How does infection occur in humans?**	**1 (1)**	**89 (91.8)**	**7 (7.2)**	
Work area				
Rural-semi-urban (n=27)	0 (0)	26 (96.3)	1 (3.7)	0.86
Urban (n=70)	1 (1.4)	63 (90)	6 (8.6)	
Work area				
Public sector (n=14)	0 (0)	13 (92.9)	1 (7.1)	
Private sector (n=81)	1 (1.2)	74 (91.4)	6 (7.4)	0.81
Unemployed (n=2)	0 (0)	2 (100)	0 (0)	
Private sector				
Small animals (n=39)	1 (2.6)	35 (89.7)	3 (7.7)	
Large animals (n=37)	0 (0)	36 (97.3)	1 (2.7)	**0.04**
Company (n=4)	0 (0)	3 (75)	1 (25)	
Technology centre (n=1)	0 (0)	0 (0)	1 (100)	

Table S5. Knowledge about forms of presentation and clinical signs in animals of the disease of survey veterinary participants from the Province of Malaga (October-December 2024). The frequency distributions and percentages obtained were determined and compared based on the demographic characteristics of the respondents, using non-parametric tests (Chi-square, Fisher's exact test, Kruskal-Wallis, and Mann-Whitney U, depending on the nature of the variable) ($\alpha = 0.05$), applying the Bonferroni correction in the case of pairwise multiple comparisons.

Total participants (n=97)				
**Question**	**Correct n (%)**	**Partially correct n (%)**	**Incorrect or I do not know n (%)**	*P* Value
**What is the most common form of presentation in animals?**	**52 (53.6)**	**-**	**45 (46.4)**	
Work area				
Rural-semi-urban (n=27)	13 (48.2)	-	14 (51.8)	0.65
Urban (n=70)	39 (55.7)	-	31 (44.3)	
Work sector				
Public sector (n=14)	9 (64.3)	-	5 (35.7)	
Private sector (n=81)	43 (53.1)	-	38 (46.9)	0.22
Unemployed (n=2)	0 (0)	-	2 (100)	
Private sector				
Small animals (n=39)	20 (51.3)	-	19 (48.7)	
Large animals (n=37)	21 (56.8)	-	16(43.2)	
Company (n=4)	1 (25)	-	3 (75)	0.49
Technology centre (n=1)	1 (100)	-	0 (0)	
**What are the most common clinical signs in sick animals?**	**40 (41.2)**	**-**	**57 (58.8)**	
Work area				
Rural-semi-urban (n=27)	8 (29.6)	-	19 (70.4)	0.14
Urban (n=70)	32 (45.7)	-	38 (54.3)	
Work sector				
Public sector (n=14)	5 (35.7)	-	9 (64.3)	
Private sector (n=81)	34 (42)	-	47 (58)	0.87
Unemployed (n=2)	1 (50)	-	1 (50)	
Private sector				
Small animals (n=39)	17 (43.6)	-	22 (56.4)	
Large animals (n=37)	15 (40.5)	-	22 (59.5)	0.5
Company (n=4)	1 (25)	-	3 (75)	
Technology centre (n=1)	1 (100)	-	0 (0)	

Table S6. Knowledge about forms of presentation and clinical signs in humans of the disease of survey veterinary participants from the Province of Malaga (October-December 2024). The frequency distributions and percentages obtained were determined and compared based on the demographic characteristics of the respondents, using non-parametric tests (Chi-square, Fisher's exact test, Kruskal-Wallis, and Mann-Whitney U, depending on the nature of the variable) (α = 0.05), applying the Bonferroni correction in the case of pairwise multiple comparisons.

Total participants (n=97)				
**Question**	**Correct n (%)**	**Partially correct n (%)**	**Incorrect or I do not know n (%)**	*P* Value
**What is the most common form of presentation in humans?**	**81 (83.5)**	**-**	**16 (16.5)**	
Work area				
Rural-semi-urban (n=27)	22 (81.5)	-	5 (18.5)	0.76
Urban (n=70)	59 (84.3)	-	11 (15.7)	
Work sector				
Public sector (n=14)	10 (71.4)	-	4 (28.6)	
Private sector (n=81)	70 (86.4)	-	11 (13.6)	0.16
Unemployed (n=2)	1 (50)	-	1 (50)	
Private sector				
Small animals (n=39)	35 (89.7)	-	4 (10.3)	
Large animals (n=37)	31 (83.8)	-	6 (16.2)	0.75
Company (n=4)	3 (75)	-	1 (25)	
Technology centre (n=1)	1 (100)	-	0 (0)	
**What are the most common clinical signs in sick people?**	**10 (10.3)**	**77 (79.4)**	**10 (10.3)**	
Work area				
Rural-semi-urban (n=27)	1 (3.7)	24 (88.9)	2 (7.4)	
Urban (n=70)	9 (12.9)	53 (75.7)	8 (11.4)	0.53
Work sector				
Public sector (n=14)	1 (7.1)	12 (85.8)	1 (7.1)	
Private sector (n=81)	9 (11.1)	64 (79)	8 (9.9)	0.23
Unemployed (n=2)	0 (0)	1 (50)	1 (50)	
Private sector				
Small animals (n=39)	5 (12.8)	30 (76.9)	4 (10.3)	
Large animals (n=37)	4 (10.8)	31 (83.8)	2 (5.4)	0.25
Company (n=4)	0 (0)	3 (75)	1 (25)	
Technology centre (n=1)	0 (0)	0 (0)	1 (100)	

Table S7. Knowledge about diagnosis and prevention of the disease of survey veterinary participants from the Province of Malaga (October-December 2024). The frequency distributions and percentages obtained were determined and compared based on the demographic characteristics of the respondents, using non-parametric tests (Chi-square, Fisher's exact test, Kruskal-Wallis, and Mann-Whitney U, depending on the nature of the variable) (α = 0.05), applying the Bonferroni correction in the case of pairwise multiple comparisons.

Total participants (n=97)				
**Question**	**Correct n (%)**	**Partially correct n (%)**	**Incorrect or I do not know n (%)**	*P* Value
**Recommended diagnostic techniques in animals**	**30 (30.9)**	**56 (57.7)**	**11 (11.4)**	
Work area				
Rural-semi-urban (n=27)	5 (18.5)	18 (66.7)	4 (14.8)	0.25
Urban (n=70)	25 (35.7)	38 (54.3)	7 (10)	
Work sector				
Public sector (n=14)	4 (28.6)	8 (57.1)	2 (14.3)	
Private sector (n=81)	25 (30.9)	48 (59.3)	8 (9.8)	0.36
Unemployed (n=2)	1 (50)	0 (0)	1 (50)	
Private sector				
Small animals (n=39)	14 (35.9)	23 (59)	2 (5.1)	
Large animals (n=37)	11 (29.7)	23 (62.2)	3 (8.1)	**0.002**
Company (n=4)	0 (0)	1 (25)	3 (75)	
Technology centre (n=1)	0 (0)	1 (100)	0 (0)	
**Main biosecurity measures on livestock farms**	**20 (20.6)**	**70 (72.2)**	**7 (7.2)**	
Work area				
Rural-semi-urban (n=27)	7 (25.9)	18 (66.7)	2 (7.4)	
Urban (n=70)	13 (18.6)	52 (74.3)	5 (7.1)	0.33
Work sector				
Public sector (n=14)	2 (14.3)	10 (71.4)	2 (14.3)	
Private sector (n=81)	14 (17.3)	63 (77.8)	4 (4.9)	0.1
Unemployed (n=2)	0 (0)	1 (50)	1 (50)	
Private sector				
Small animals (n=39)	10 (25.6)	27 (69.2)	2 (5.2)	
Large animals (n=37)	3 (8.1)	33 (89.2)	1 (2.7)	0.13
Company (n=4)	1 (25)	2 (50)	1 (25)	
Technology centre (n=1)	0 (0)	1 (100)	0 (0)	
**Main measures to prevent human infection**	**2 (2.1)**	**88 (90.7)**	**7 (7.2)**	
Work area				
Rural-semi-urban (n=27)	0 (0)	25 (92.6)	2 (7.4)	0.84
Urban (n=70)	2 (2.9)	63 (90)	5 (7.1)	
Work sector				
Public sector (n=14)	0 (0)	12 (85.7)	2 (14.3)	
Private sector (n=81)	2 (2.5)	75 (92.6)	4 (4.9)	0.22
Unemployed (n=2)	0 (0)	1 (50)	1 (50)	
Private sector				
Small animals (n=39)	2 (5.1)	35 (89.8)	2 (5.1)	
Large animals (n=37)	0 (0)	36 (97.3)	1 (2.7)	0.68
Company (n=4)	0 (0)	3 (75)	1 (25)	
Technology centre (n=1)	0 (0)	1 (100)	0 (0)	

Table S8. Knowledge of the disease in Spain of survey veterinary participants from the Province of Malaga (October-December 2024). The frequency distributions and percentages obtained were determined and compared based on the demographic characteristics of the respondents, using non-parametric tests (Chi-square, Fisher's exact test, Kruskal-Wallis, and Mann-Whitney U, depending on the nature of the variable) (α = 0.05), applying the Bonferroni correction in the case of pairwise multiple comparisons.

Total participants (n=97)				
**Question**	**Correct n (%)**	**Partially correct n (%)**	**Incorrect or I do not know n (%)**	*P* Value
**Is it a Notifiable Disease?**	**69 (71.1)**	**-**	**28 (28.9)**	
Work area				
Rural-semi-urban (n=27)	20 (74.1)	-	7 (25.9)	0.69
Urban (n=70)	49 (70)	-	21 (30)	
Work sector				
Public sector (n=14)	8 (57.1)	-	6 (42.9)	
Private sector (n=81)	60 (74.1)	-	21 (25.9)	0.35
Unemployed (n=2)	1 (50)	-	1 (50)	
Private sector				
Small animals (n=39)	30 (76.9)	-	9 (23.1)	
Large animals (n=37)	29 (78.4)	-	8 (21.6)	
Company (n=4)	1 (25)	-	3 (75)	**0.04**
Technology centre (n=1)	0 (0)	-	1 (100)	
**What is the typical presentation of the disease in humans?**	**36 (37.1)**	**-**	**61 (62.9)**	
Work area				
Rural-semi-urban (n=27)	12 (44.4)	-	15 (55.6)	0.35
Urban (n=70)	24 (34.3)	-	46 (65.7)	
Work sector				
Public sector (n=14)	3 (21.4)	-	11 (78.6)	
Private sector (n=81)	33 (40.7)	-	48 (59.3)	0.21
Unemployed (n=2)	0 (0)	-	2 (100)	
Private sector				
Small animals (n=39)	17 (43.6)	-	22 (56.4)	
Large animals (n=37)	16 (43.2)	-	21 (56.8)	0.3
Company (n=4)	0 (0)	-	4 (100)	
Technology centre (n=1)	0 (0)	-	1 (100)	
**During what period of the year do most human cases occur?**	**75 (77.3)**	**-**	**22 (22.7)**	
Work area				
Rural-semi-urban (n=27)	19 (70.4)	-	8 (29.6)	0.31
Urban (n=70)	56 (80)	-	14 (20)	
Work sector				
Public sector (n=14)	8 (57.1)	-	6 (42.9)	
Private sector (n=81)	65 (80.3)	-	16 (19.7)	0.12
Unemployed (n=2)	2 (100)	-	0 (0)	
Private sector				
Small animals (n=39)	34 (87.2)	-	5 (12.8)	
Large animals (n=37)	31 (83.8)	-	6 (16.2)	**0.001**
Company (n=4)	0 (0)	-	4 (100)	
Technology centre (n=1)	0 (0)	-	1 (100)	

Table S9. Classification of knowledge of survey veterinary participants from the Province of Malaga (October-December 2024). The frequency distributions and percentages obtained were determined and compared based on the demographic characteristics of the respondents, using non-parametric tests (Chi-square, Fisher's exact test, Kruskal-Wallis, and Mann-Whitney U, depending on the nature of the variable) ($\alpha = 0.05$), applying the Bonferroni correction in the case of pairwise multiple comparisons.

**Total participants (n=97)**				
**General knowledge**	**Poor n (%)**	**Good n (%)**	**Very good n (%)**	P Value
Gender				
Male (n=54)	31 (57.4)	17 (31.5)	6 (11.1)	
Female (n=42)	22 (52.4)	15 (35.7)	5 (11.9)	0.69
Other (n=1)	0 (0)	1 (100)	0 (0)	
Age				
22 - 30 years (n=10)	4 (40)	5 (50)	1 (10)	
31 - 40 years (n=32)	19 (59.4)	9 (28.1)	4 (12.5)	0.61
41 - 50 years (n=35)	21 (60)	12 (34.3)	2 (5.7)	
Over 50 years old (n=20)	9 (45)	7 (35)	4 (20)	
Work sector				
Public sector (n=14)	6 (42.9)	8 (57.1)	0 (0)	
Private sector (n=81)	46 (56.8)	24 (29.6)	11 (13.6)	0.25
Unoccupied (n=2)	1 (50)	1 (50)	0 (0)	
**Total**	**53 (54.6)**	**33 (34)**	**11 (11.4)**	

Table S10. Perception of the information received through official channels about Q Fever of survey veterinary participants from the Province of Malaga (October-December 2024). The frequency distributions and percentages obtained were determined and compared based on the demographic characteristics of the respondents, using non-parametric tests (Chi-square, Fisher's exact test, Kruskal-Wallis, and Mann-Whitney U, depending on the nature of the variable) ($\alpha = 0.05$), applying the Bonferroni correction in the case of pairwise multiple comparisons.

**Total participants (n=97)**						
**Do you think that official media (ministry, regional government, veterinary associations, etc.) sufficiently emphasize the importance of this disease as an occupational zoonosis? Rate their performance in this regard from 1 to 5.**						
	**n (%)**					
**Score 1 (P1)**	49 (51.2)					
**Score 2 (P2)**	42 (42.3)					
**Score 3 (P3)**	5 (5.3)					
**Score 4 (P4)**	0 (0)					
**Score 5 (P5)**	1 (1.2)					
	**P1 n (%)**	**P2 n (%)**	**P3 n (%)**	**P4 n (%)**	**P5 n (%)**	*P* Value
Age						
22 - 30 years (n=10)	6 (60)	4 (40)	0 (0)	0 (0)	0 (0)	
31 - 40 years (n=32)	15 (46.9)	15 (46.9)	2 (6.2)	0 (0)	0 (0)	0.69
41 - 50 years (n=35)	15 (42.9)	17 (48.6)	2 (5.7)	0 (0)	1 (2.8)	
Over 50 years old (n=20)	14 (70)	5 (25)	1 (5)	0 (0)	0 (0)	
Knowledge						
Poor (n=53)	29 (54.7)	21 (39.6)	3 (5.7)	0 (0)	0 (0)	0.56
Good (n=33)	14 (42.4)	17 (51.6)	1 (3)	0 (0)	1 (3)	
Very good (n=11)	7 (63.6)	3 (27.3)	1 (9.1)	0 (0)	0 (0)	
Work sector						
Public sector (n=14)	10 (71.4)	4 (28.6)	0 (0)	0 (0)	0 (0)	0.78
Private sector (n=81)	39 (48.2)	36 (44.4)	5 (6.2)	0 (0)	1 (1.2)	
Unemployed (n=2)	1 (50)	1 (50)	0 (0)	0 (0)	0 (0)	

Table S11. Perception of the reliability of information sources about Q Fever of survey veterinary participants from the Province of Malaga (October-December 2024). The frequency distributions and percentages obtained were determined and compared based on the demographic characteristics of the respondents, using non-parametric tests (Chi-square, Fisher's exact test, Kruskal-Wallis, and Mann-Whitney U, depending on the nature of the variable) (α = 0.05), applying the Bonferroni correction in the case of pairwise multiple comparisons.

**Total participants (n=97)**			
**Do you consider you have a reliable source of information regarding the protocol for handling Q fever outbreaks in animals?**			
	**Yes n (%)**	**No n (%)**	*P* Value
Age			
22 - 30 years (n=10)	1 (10)	9 (90)	0.28
31 - 40 years (n=32)	1 (3.1)	31 (96.9)	
41 - 50 years (n=35)	4 (11.4)	31 (88.6)	
Over 50 years old (n=20)	4 (20)	16 (80)	
Knowledge			
Poor (n=53)	3 (5.6)	50 (94.4)	0.17
Good (n=33)	6 (18.2)	27 (81.8)	
Very good (n=11)	1 (9.1)	10 (90.9)	
Work sector			
Public sector (n=14)	1 (7.1)	13 (92.9)	0.17
Private sector (n=81)	8 (9.9)	73 (90.1)	
Unemployed (n=2)	1 (50)	1 (50)	

Table S12. Perception of the performance of diagnostic tests of survey veterinary participants from the Province of Malaga (October-December 2024). The frequency distributions and percentages obtained were determined and compared based on the demographic characteristics of the respondents, using non-parametric tests (Chi-square, Fisher's exact test, Kruskal-Wallis, and Mann-Whitney U, depending on the nature of the variable) (α = 0.05), applying the Bonferroni correction in the case of pairwise multiple comparisons.

**Total participants (n=97)**						
**In your opinion, should veterinarians undergo diagnostic testing for Q fever if the disease is detected in their work area?**						
					**n (%)**	
Yes, all of them					20 (20.6)	
No, none					0 (0)	
Only veterinarians in contact with animals from the outbreak					24 (24.7)	
Only veterinarians in contact with animals susceptible to infection					48 (49.5)	
Do not know					5 (5.2)	
	**All n (%)**	**None n (%)**	**Contact animal's outbreak n (%)**	**Contact with susceptible animals n (%)**	**Do not know n (%)**	*P* Value
Rural-semi-urban (n=27)	6 (22.2)	0 (0)	7 (25.9)	14 (51.9)	0 (0)	0.56
Urban (n=70)	14 (20)	0 (0)	17 (24.3)	34 (48.6)	5 (7.1)	
Public sector (n=14)	2 (14.3)	0 (0)	3 (21.4)	7 (50)	2 (14.3)	**0.04**
Private sector (n=81)	18 (22.2)	0 (0)	21 (25.9)	40 (49.4)	2 (2.5)	
Unemployed (n=2)	0 (0)	0 (0)	0 (0)	1 (50)	1 (50)	
Private sector						
Small animals (n=39)	10 (25.6)	0 (0)	9 (23.1)	18 (46.1)	2 (5.2)	0.45
Large animals (n=37)	6 (16.2)	0 (0)	12 (32.4)	19 (51.4)	0 (0)	
Company (n=4)	1 (25)	0 (0)	0 (0)	3 (75)	0 (0)	
Technology centre (n=1)	1 (100)	0 (0)	0 (0)	0 (0)	0 (0)	

Table S13. Perception of the risk of having pets of survey veterinary participants from the Province of Malaga (October-December 2024). The frequency distributions and percentages obtained were determined and compared based on the demographic characteristics of the respondents, using non-parametric tests (Chi-square, Fisher's exact test, Kruskal-Wallis, and Mann-Whitney U, depending on the nature of the variable) (α = 0.05), applying the Bonferroni correction in the case of pairwise multiple comparisons.

**Total participants (n=97)**						
**Do you think having any of these animals as pets could put you at additional risk of contracting Q Fever?**						
	**n (%)**					
Dog and cat	63 (64.9)					
Birds	9 (9.3)					
Rabbit and/or rodents	4 (4.1)					
Reptiles	4 (4.1)					
Do not know	17 (17.6)					
	**Dog and cat n (%)**	**Birds n (%)**	**Rabbit n (%)**	**Reptile n (%)**	**I do not know n (%)**	*P* Value
Age						
22 - 30 years (n=10)	8 (80)	0 (0)	1 (10)	0 (0)	1 (10)	
31 - 40 years (n=32)	29 (90.6)	2 (6.3)	0 (0)	0 (0)	1 (3.1)	0.06
41 - 50 years (n=35)	27 (77.1)	0 (0)	0 (0)	0 (0)	8 (22.9)	
Over 50 years old (n=20)	11 (55)	0 (0)	1 (5)	1 (5)	7 (35)	
Knowledge						
Poor (n=53)	37 (69.8)	1 (1.9)	2 (3.8)	0 (0)	13 (24.51)	
Good (n=33)	29 (87.9)	0 (0)	0 (0)	0 (0)	4 (12.1)	**0.02**
Very good (n=11)	9 (81.8)	1 (9.1)	0 (0)	1 (9.1)	0 (0)	
Work sector						
Public sector (n=14)	9 (64.3)	0 (0)	1 (7.1)	0 (0)	4 (28.6)	
Private sector (n=81)	65 (80.3)	2 (2.5)	0 (0)	1 (1.2)	13 (16)	**0.01**
Unemployed (n=2)	1 (50)	0 (0)	1 (50)	0 (0)	0 (0)	

Table S14. Attitude toward the use of protective equipment of survey veterinary participants from the Province of Malaga (October-December 2024). The frequency distributions and percentages obtained were determined and compared based on the demographic characteristics of the respondents, using non-parametric tests (Chi-square, Fisher's exact test, Kruskal-Wallis, and Mann-Whitney U, depending on the nature of the variable) (α = 0.05), applying the Bonferroni correction in the case of pairwise multiple comparisons.

**Total participants (n=97)**						
**Do you use appropriate protective equipment when handling animals suspected of having Q Fever, their carcasses, tissues or fluids?**						
	**n (%)**					
**Always**	43 (44.3)					
**Most of the time**	34 (35.1)					
**Sometimes**	4 (4.1)					
**Rarely**	6 (6.2)					
**Never**	10 (10.3)					
	**Always n (%)**	**Most of the time n (%)**	**Sometimes n (%)**	**Rarely n (%)**	**Never n (%)**	*P* Value
Age						
22 - 30 years (n=10)	4 (40)	3 (30)	0 (0)	1 (10)	2 (20)	
31 - 40 years (n=32)	18 (56.3)	12 (37.4)	2 (6.3)	0 (0)	0 (0)	
41 - 50 years (n=35)	18 (51.4)	14 (40)	0 (0)	0 (0)	3 (8.6)	**0.001**
Over 50 years old (n=20)	3 (15)	5 (25)	2 (10)	5 (25)	5 (25)	
Rural-semi-urban (n=27)	14 (51.9)	7 (25.9)	0 (0)	3 (11.1)	3 (11.1)	
Urban (n=70)	29 (41.4)	27 (38.6)	4 (5.7)	3 (4.3)	7 (10)	0.35
Public sector (n=14)	7 (50)	3 (21.4)	0 (0)	1 (7.2)	3 (21.4)	
Private sector (n=81)	36 (44.4)	31 (38.3)	4 (4.9)	5 (6.2)	5 (6.2)	**0.004**
Unemployed (n=2)	0 (0)	0 (0)	0 (0)	0 (0)	2 (100)	
Private sector						
Small animals (n=39)	16 (41)	15 (38.5)	4 (10.3)	1 (2.5)	3 (7.7)	
Large animals (n=37)	18 (48.7)	13 (35.1)	0 (0)	4 (10.8)	2 (5.4)	0.58
Company (n=4)	1 (25)	3 (75)	0 (0)	0 (0)	0 (0)	
Technology centre (n=1)	1 (100)	0 (0)	0 (0)	0 (0)	0 (0)	

Table S15. Attitude toward vaccination status against Q Fever of survey veterinary participants from the Province of Malaga (October-December 2024). The frequency distributions and percentages obtained were determined and compared based on the demographic characteristics of the respondents, using non-parametric tests (Chi-square, Fisher's exact test, Kruskal-Wallis, and Mann-Whitney U, depending on the nature of the variable) (α = 0.05), applying the Bonferroni correction in the case of pairwise multiple comparisons.

**Total participants (n=97)**					
**Are you vaccinated against Q Fever?**					
	**n (%)**				
Yes	0 (0)				
No	29 (29.9)				
In case of outbreaks	2 (2.1)				
I do not know if there is a vaccine.	66 (68)				
	**Yes n (%)**	**No n (%)**	**In case of outbreaks n (%)**	**I do not know if there is a vaccine n (%)**	P Value
Age					
22 - 30 years (n=10)	0 (0)	5 (50)	0 (0)	5 (50)	
31 - 40 years (n=32)	0 (0)	8 (25)	1 (3.1)	23 (71.9)	
41 - 50 years (n=35)	0 (0)	9 (25.7)	0 (0)	26 (74.3)	0.56
Over 50 years old (n=20)	0 (0)	7 (35)	1 (5)	12 (60)	
Rural-semi-urban (n=27)	0 (0)	7 (25.9)	1 (3.7)	19 (70.4)	0.69
Urban (n=70)	0 (0)	22 (31.4)	1 (1.4)	47 (67.2)	
Public sector (n=14)	0 (0)	4 (28.6)	0 (0)	10 (71.4)	
Private sector (n=81)	0 (0)	24 (29.6)	2 (2.5)	55 (67.9)	0.93
Unemployed (n=2)	0 (0)	1 (50)	0 (0)	1 (50)	
Private sector					
Small animals (n=39)	0 (0)	12 (30.8)	1 (2.6)	26 (66.6)	
Large animals (n=37)	0 (0)	11 (29.7)	1 (2.7)	25 (67.6)	0.99
Company (n=4)	0 (0)	1 (25)	0 (0)	3 (75)	
Technology centre (n=1)	0 (0)	0 (0)	0 (0)	1 (100)	

Table S16. Attitude toward taking preventive medication authorized by Ministry of Labour's Good Practices Guide for occupational zoonoses (NTP411) of survey veterinary participants from the Province of Malaga (October-December 2024). The frequency distributions and percentages obtained were determined and compared based on the demographic characteristics of the respondents, using non-parametric tests (Chi-square, Fisher's exact test, Kruskal-Wallis, and Mann-Whitney U, depending on the nature of the variable) ($\alpha = 0.05$), applying the Bonferroni correction in the case of pairwise multiple comparisons.

**Total participants (n=97)**			
**Did you know that the Ministry of Labour's Good Practices Guide for occupational zoonoses (NTP411) allows immunoprophylaxis (vaccination) and chemoprophylaxis (antimicrobials) in cases where personal protective equipment (PPE) is not possible?**			
		**n (%)**	
	Yes	2 (2.1)	
	No	95 (97.9)	
	**Yes n (%)**	**No n (%)**	P Value
Age			
22 - 30 years (n=10)	1 (10)	9 (90)	
31 - 40 years (n=32)	0 (0)	32 (100)	
41 - 50 years (n=35)	0 (0)	35 (100)	0.14
Over 50 years old (n=20)	1 (5)	19 (95)	
Rural-semi-urban (n=27)	0 (0)	27 (100)	
Urban (n=70)	2 (2.9)	68 (97.1)	0.37
Public sector (n=14)	0 (0)	14 (100)	
Private sector (n=81)	1 (1.2)	80 (98.8)	**0.001**
Unemployed (n=2)	1 (50)	1 (50)	
Private sector			
Small animals (n=39)	1 (2.6)	38 (97.4)	
Large animals (n=37)	0 (0)	37 (100)	0.77
Company (n=4)	0 (0)	4 (100)	
Technology centre (n=1)	0 (0)	1 (100)	

Table S17. Attitude toward staying informed about Q Fever in humans of survey veterinary participants from the Province of Malaga (October-December 2024). The frequency distributions and percentages obtained were determined and compared based on the demographic characteristics of the respondents, using non-parametric tests (Chi-square, Kruskal-Wallis, and Mann-Whitney U, depending on the nature of the variable) ($\alpha = 0.05$), applying the Bonferroni correction in the case of pairwise multiple comparisons.

**Total participants (n=97)**				
**Are you up to date on the human situation of Q Fever in Spain?**				
		**n (%)**		
Yes		12 (12.4)		
No		39 (40.2)		
In case of outbreaks		46 (47.4)		
	**Yes n (%)**	**No n (%)**	**In case of outbreaks n (%)**	P Value
Age				
22 - 30 years (n=10)	0 (0)	4 (40)	6 (60)	
31 - 40 years (n=32)	8 (25)	7 (21.9)	17 (53.1)	
41 - 50 years (n=35)	3 (8.6)	16 (45.7)	16 (45.7)	**0.047**
Over 50 years old (n=20)	1 (5)	12 (60)	7 (35)	
Rural-semi-urban (n=27)	3 (11.1)	9 (33.3)	15 (55.6)	
Urban (n=70)	9 (12.9)	30 (42.9)	31 (44.3)	0.6
Public sector (n=14)	0 (0)	7 (50)	7 (50)	
Private sector (n=81)	12 (14.8)	31 (38.3)	38 (46.9)	0.58
Unemployed (n=2)	0 (0)	1 (50)	1 (50)	
Private sector				
Small animals (n=39)	5 (12.8)	17 (43.6)	17 (43.6)	
Large animals (n=37)	7 (18.9)	11 (29.7)	19 (51.4)	0.66
Company (n=4)	0 (0)	2 (50)	2 (50)	
Technology centre (n=1)	0 (0)	1 (100)	0 (0)	

Table S18. Attitude toward staying informed about Q Fever in animals of survey veterinary participants from the Province of Malaga (October-December 2024). The frequency distributions and percentages obtained were determined and compared based on the demographic characteristics of the respondents, using non-parametric tests (Chi-square, Kruskal-Wallis, and Mann-Whitney U, depending on the nature of the variable) ($\alpha = 0.05$), applying the Bonferroni correction in the case of pairwise multiple comparisons.

**Total participants (n=97)**				
**Are you up to date on the Q Fever situation in animals in Spain?**				
			**n (%)**	
Yes			29 (29.9)	
No			24 (24.7)	
In case of outbreaks			44 (45.4)	
	**Yes**<br>**n (%)**	**No**<br>**n (%)**	**In case of outbreaks**<br>**n (%)**	P Value
Age				
22 - 30 years (n=10)	2 (20)	2 (30)	5 (50)	
31 - 40 years (n=32)	12 (37.5)	1 (3.1)	19 (59.4)	
41 - 50 years (n=35)	11 (31.4)	9 (25.7)	15 (42.9)	**0.005**
Over 50 years old (n=20)	4 (20)	11 (55)	5 (25)	
Rural-semi-urban (n=27)	7 (25.9)	7 (25.9)	13 (48.2)	
Urban (n=70)	22 (31.4)	17 (24.3)	31 (44.3)	0.86
Public sector (n=14)	2 (14.2)	6 (42.9)	6 (42.9)	
Private sector (n=81)	27 (33.3)	17 (21)	37 (45.7)	0.29
Unemployed (n=2)	0 (0)	1 (50)	1 (50)	
Private sector				
Small animals (n=39)	13 (33.3)	8 (20.5)	18 (46.2)	
Large animals (n=37)	14 (37.8)	6 (16.2)	17 (46)	0.29
Company (n=4)	0 (0)	2 (50)	2 (50)	
Technology centre (n=1)	0 (0)	1 (100)	0 (0)	

Supplementary B. Survey

Knowledge, perception, and attitude toward Q Fever among veterinarians in the province of Malaga

Fields marked with * are required.

This study was conducted by the PAIDI AGR-256 group of the Department of Animal Health at the University of Córdoba. Researchers were Dr. Belén Huerta Lorenzo and Dr. Rafael Jesús Astorga Márquez.

1. OBJECTIVE OF THE STUDY:
To assess the training, perception and attitude of veterinarians in the province of Malaga regarding Q Fever , as a population at risk, as well as their vaccination status against the disease.

In compliance with the Data Protection and Digital Rights Guarantee Act (LOPDGDD) 2018 and the General Data Protection Regulation (GDPR) 2022, we inform you that this survey is confidential and that all information collected, both personal and professional, will be encrypted and safeguarded by the study managers until its completion, after which it will be deleted. The data will be processed exclusively for research purposes and will not be shared with third parties.

2. ESTIMATED RESPONSE TIME: 15 minutes
If any questions remain unanswered, the platform will require you to review your questionnaire. Please make sure you answer all mandatory questions for the questionnaire to be completed.

We appreciate your sharing with other registered veterinarian in the province of Malaga.

THANK YOU VERY MUCH FOR YOUR COLLABORATION

3. DEMOGRAPHIC DATA

* Question **3.1. Gender**

☐ Male

☐ Female

☐ Another option

* Question **3.2. Age**

☐ 22-30 years old

☐ 31-40 years old

☐ 41-50 years old

☐ Over 50 years old

* Question **3.3. Higher level of education**

- Graduate in Veterinary Medicine
- Doctor of Veterinary Medicine

Question **3.4. Other qualifications (Master's, Bachelor's or Degrees)**

* Question **3.5. Current situation.** You may select more than one option.

- Public sector
- Private sector
- Unemployed

Question **3.6.** (Answer only if you selected "Public Sector" in the previous question) **Usual areas of work.** *You can select more than one option.*

- Ministry or Department of Health
- Ministry or Council of Agriculture
- Technology or Research Center
- University
- ESO or Vocational Training Center
- Other

Question **3.7. If you selected "Other," please indicate:**

Question **3.8.** (Answer only if you selected "Private sector" in question 3.5.) **Usual areas of work.** *You can select more than one option.*

- Small Animal Clinic
- Large Animal Clinic
- Animal production
- Food company
- Environmental company
- Technology or research center
- Laboratory
- Other

Question **3.9. If you selected "Other," please indicate:**

* Question **3.10. Usual work area**

- ○ Rural (<2,000 inhabitants)
- ○ Semi-urban (2,000-10,000 inhabitants)
- ○ Urban (>10,000 inhabitants)

4. GENERAL KNOWLEDGE OF THE DISEASE

* Question **4.1. Is Q Fever a zoonosis?**

- ◉ Yes
- ○ No
- ○ I do not know

* Question **4.2. Do you know the nature of its etiological agent?**

- ○ Fungus
- ◉ Bacterium
- ○ Virus
- ○ Parasite
- ○ I do not know

* Question **4.3. In which areas is the disease endemic in the animal population?** *You can select more than one option.*

- ☑ Africa
- ☑ Europe
- ☑ America
- ☑ Asia
- ☐ Oceania
- ☐ I do not know

* Question **4.4. Which domestic species are most susceptible?** *You can select more than one option.*

- ☐ Pig
- ☑ Ruminants (cow, sheep and goat)
- ☐ Dog and cat
- ☐ Horse
- ☐ I do not know

* Question **4.5. What are its wild reservoirs?** *You can select more than one option.*

- ☑ Migratory birds
- ☐ Rodents
- ☑ Deer
- ☑ Wild boar
- ☐ I do not know

* Question **4.6. How does infection occur in animals?** *You can select more than one option.*

- ☑ Tick bite

- [ ] Genital route
- [x] Inhalation of contaminated aerosols and dust
- [x] Ingestion of water and food contaminated with feces, slurry and manure
- [x] Transplacental transmission
- [ ] I do not know

* Question **4.7. How does infection occur in humans?** *You can select more than one option.*

- [x] Inhalation of contaminated aerosols and dust
- [x] Tick bite
- [x] Consumption of water and food contaminated with animal secretions
- [ ] Contact with infected people, their fluids or tissues
- [x] Contact with infected animals, their carcasses, fluids and tissues
- [ ] Puncture with contaminated objects
- [ ] Consumption of animal products from infected subjects
- [ ] I do not know

* Question **4.8. What is the most common form of presentation in animals?**

- (•) Asymptomatic
- ( ) Acute
- ( ) Chronicle
- ( ) I do not know

* Question **4.9. What are the most common clinical signs in sick animals?** *You can select more than one option.*

- [x] Reproductive disorders, abortions and perinatal mortality
- [ ] Jaundice
- [ ] Encephalitis
- [ ] Petechiae in mucous membranes
- [ ] Generalized edema
- [ ] I do not know

* Question **4.10. What is the most common form of presentation in humans?**

- (•) Asymptomatic
- ( ) Acute
- ( ) Chronicle
- ( ) I do not know

* Question **4.11. What are the most common clinical signs in sick people?** *You can select more than one option.*

- [ ] Vomiting and diarrhea
- [x] Fatigue

- [x] Fever
- [x] Pneumonia
- [x] Endocarditis
- [ ] I do not know

* Question **4.12. What diagnostic techniques are recommended for animals?** *You can select more than one option.*

- [x] ELISA (Enzyme-linked immunosorbent assay)
- [x] PCR (Polymerase chain reaction)
- [ ] Western blot
- [ ] Microbiological culture
- [ ] I do not know

* Question **4.13. Main biosecurity measures on livestock farms to reduce the spread of the disease.** *You can select more than one option.*

- [x] Treatment of feces, slurry and manure
- [x] Vaccination
- [x] Cleaning, disinfection, pest control and rat extermination of the facilities
- [x] Isolation of females in the farrowing pen
- [x] Proper disposal of placenta and fetal remains
- [x] Control of tick infestation in animals and stables
- [x] Access control of wild and peridomestic reservoirs
- [ ] Sanitary ford
- [ ] I do not know

* Question **4.14. Main measures to prevent human infection.** *You can select more than one option.*

- [x] Wear protective clothing (long pants and sleeves), acaricides and/or authorized repellents when hiking.
- [x] Vaccination
- [x] Wear gloves and protective equipment (mask, gown/coverall, and socks) when handling animals.
- [x] Wear gloves and masks when dealing with infected people
- [x] Quarantine animals or systematically treat them with pesticides two weeks before sending them to slaughter.
- [x] Control tick infestation in animals and stables
- [ ] I do not know

<u>5. KNOWLEDGE OF THE DISEASE IN SPAIN</u>

* Question **5.1. Is it a Notifiable Disease?**

- ( ) Yes, only in animals
- ( ) Yes, only in humans

- [x] Yes, in animals and humans
- [ ] No
- [ ] I do not know

* Question **5.2. How does the disease usually present in people?**

- [x] In the form of an endemic
- [ ] In sporadic outbreaks
- [ ] Like an epidemic
- [ ] I do not know

* Question **5.3. During what period of the year do most human cases occur?**

- [ ] Autumn-winter
- [x] Spring-summer
- [ ] Both
- [ ] I do not know

6. PERCEPTION OF *Q FEVER*

* Question **6.1. Do you think official media (ministries, regional governments, veterinary associations, etc.) sufficiently emphasize the importance of this disease as an occupational zoonosis?** *Rate their performance in this regard from 1 to 5.*

Only values between 1 and 5 are allowed

* Question **6.2. Do you consider that you have a reliable source of information regarding the protocol for action in cases of Q Fever outbreaks in animals?**

- [ ] Yes
- [ ] No

* Question **6.3. In your opinion, should veterinarians undergo diagnostic testing for Q fever if the disease is detected in their work area?**

- [ ] Yes, all of them
- [ ] No, none
- [ ] Veterinarians in contact with animals from the outbreak
- [ ] Veterinarians in contact with animals susceptible to infection
- [ ] I do not know

* Question **6.4. Do you think having any of these animals as pets could put you at additional risk of contracting Q Fever ?** *You can select more than one option.*

- [ ] Dog and cat
- [ ] Birds
- [ ] Rabbit
- [ ] Guinea pig and/or hamster

- ☐ Reptiles
- ☐ I do not know

7. ATTITUDE TOWARDS *Q FEVER*

* Question **7.1. Do you use appropriate protective equipment when handling animals suspected of having Q Fever, their carcasses, tissues or fluids?**

- ○ Always
- ○ Most of the time
- ○ Sometimes
- ○ Rarely
- ○ Never

* Question **7.2. What protective measures do you usually take?** *You can select more than one option.*

- ☐ Latex gloves
- ☐ Masks
- ☐ Protective clothing (long pants, overalls, etc.)
- ☐ Repellents and/or acaricides
- ☐ Hose
- ☐ All of the above
- ☐ None

* Question **7.3. Do you get vaccinated against Q Fever ?**

- ○ Yes, periodically
- ○ No, I don't consider it necessary.
- ○ Only in cases of declared outbreaks
- ○ I do not know if there is a vaccination against Q fever.

* Question **7.4. Do you take preventative medication when handling animals that show symptoms compatible with Q fever or have tested positive for Q fever?**

- ○ Yes
- ○ No

* Question **7.5. Did you know that the Ministry of Labor's Good Practice Guide for occupational zoonoses (NTP411) allows immunoprophylaxis (vaccination) and chemoprophylaxis (antimicrobials) in cases where personal biosecurity measures (PPE) are not possible?**

- ○ Yes
- ○ No

* Question **7.6. Do you stay informed about the human situation of Q fever in Spain?**

- ○ Yes
- ○ No
- ○ Only when an outbreak is reported in the media

* Question **7.7. Do you stay informed about the Q Fever situation in animals in Spain?**

- [ ] Yes
- [ ] No
- [ ] Only when an outbreak is reported in the media
